# Supplementary material for: Microbiota derived short chain fatty acids promote histone crotonylation in the colon through histone deacetylases
Source: Nat Commun. 2018 Jan 9;9:105. doi: 10.1038/s41467-017-02651-5 (PMC5760624; doi:10.1038/s41467-017-02651-5)
Supplement: Supplementary file 1 — Supplementary Information [file 41467_2017_2651_MOESM1_ESM.pdf]

Supplementary Information Fellows et al.

Supplementary Figures 1-11

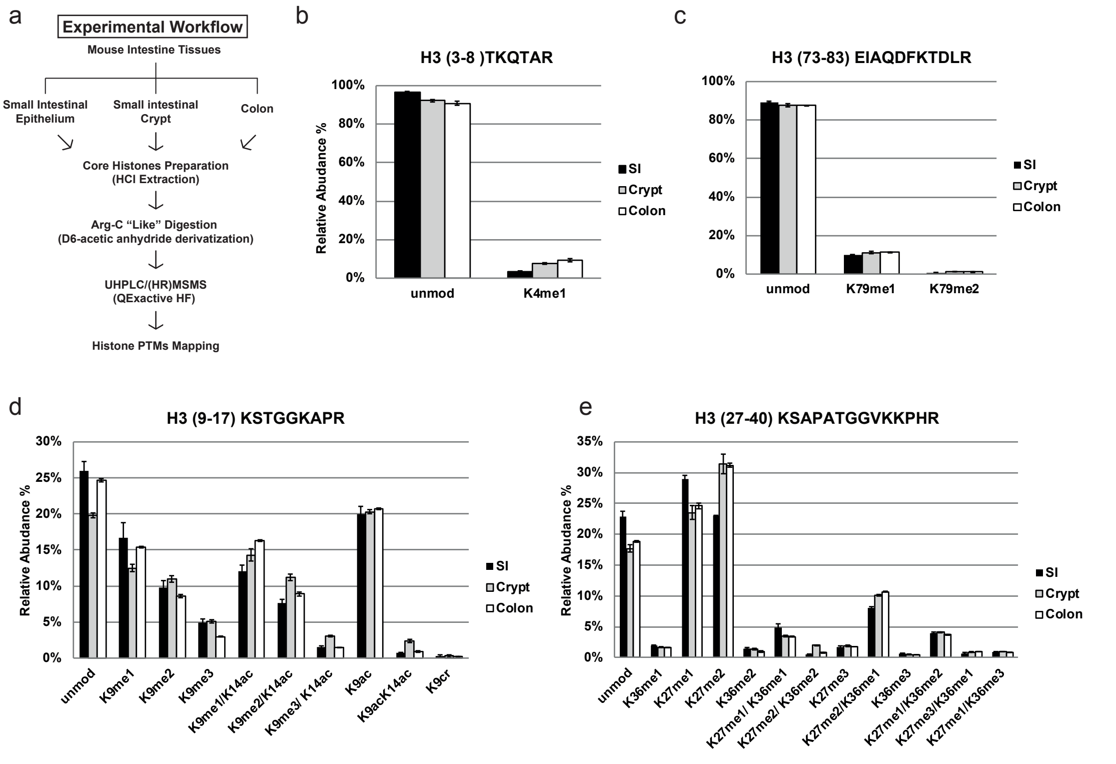

Supplementary Fig. 1. Quantification of histone H3 PTMs from intestinal epithelium. (a) Schematic representation of MS-based label-free approach using high resolution (HR)-MS/MS analysis for histone PTMs profiling in intestinal epithelium fractions **(b)** Estimated relative abundance percentage (RA%) of differentially modified (3-8) peptides bearing H3K4me1 in histones extracted from small intestinal epithelium (SI), the crypt enriched fraction (Crypt) and the colon epithelium (Colon). Peptides containing H3K4me2 and -me3 were below the detection limit. **(c)** Relative abundance percentage (RA%) of H3K79 methylation; peptides containing H3K79me3 were below the detection limit. **(d)** RA% of differentially modified peptides H3(9-17). **(e)** RA% of differentially modified H3 (27-40) peptides. For panels (b) to (e) the histone modifications with a RA% <0.1 were not reported (for a complete summary refer to Supplementary table1), n=3, error bars are standard deviation.

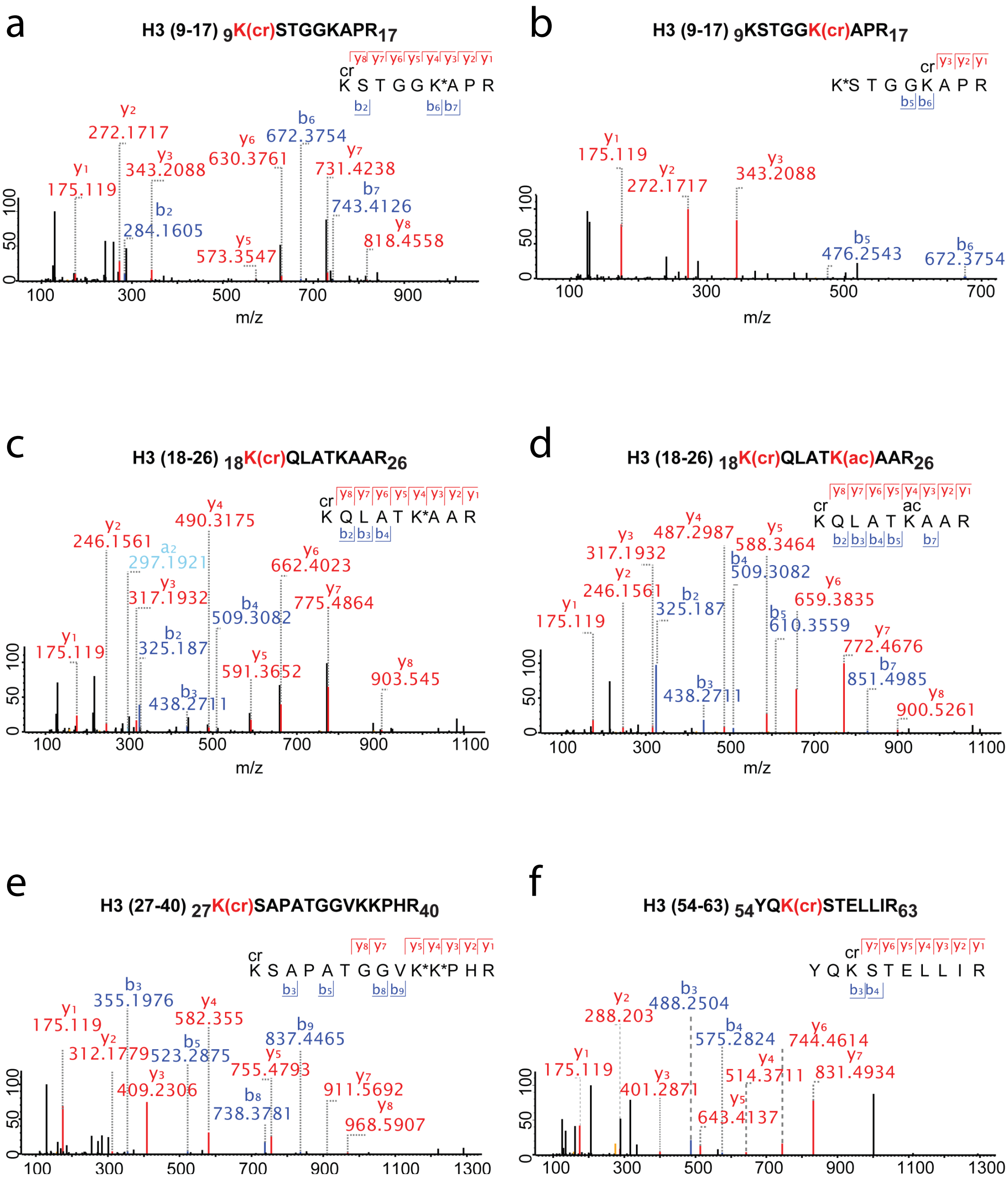

Supplementary Fig. 2. MS/MS fragmentation of crotonylated H3 peptides.

We MS/MS-annotated the peptides: H3(9-17) containing K9cr (a) and K14cr (b); H3(18-26) containing K18cr (c) and H3K18cr/K23ac (d); H3(27-40) containing K27cr (e) and H3(54-63) containing K56cr (f). For each peptide, the full annotation produced by the Viewer software within the MaxQuant suite was displayed (see Material and Method section), with matched b- and y- ions reported in blue and red colour code, respectively.

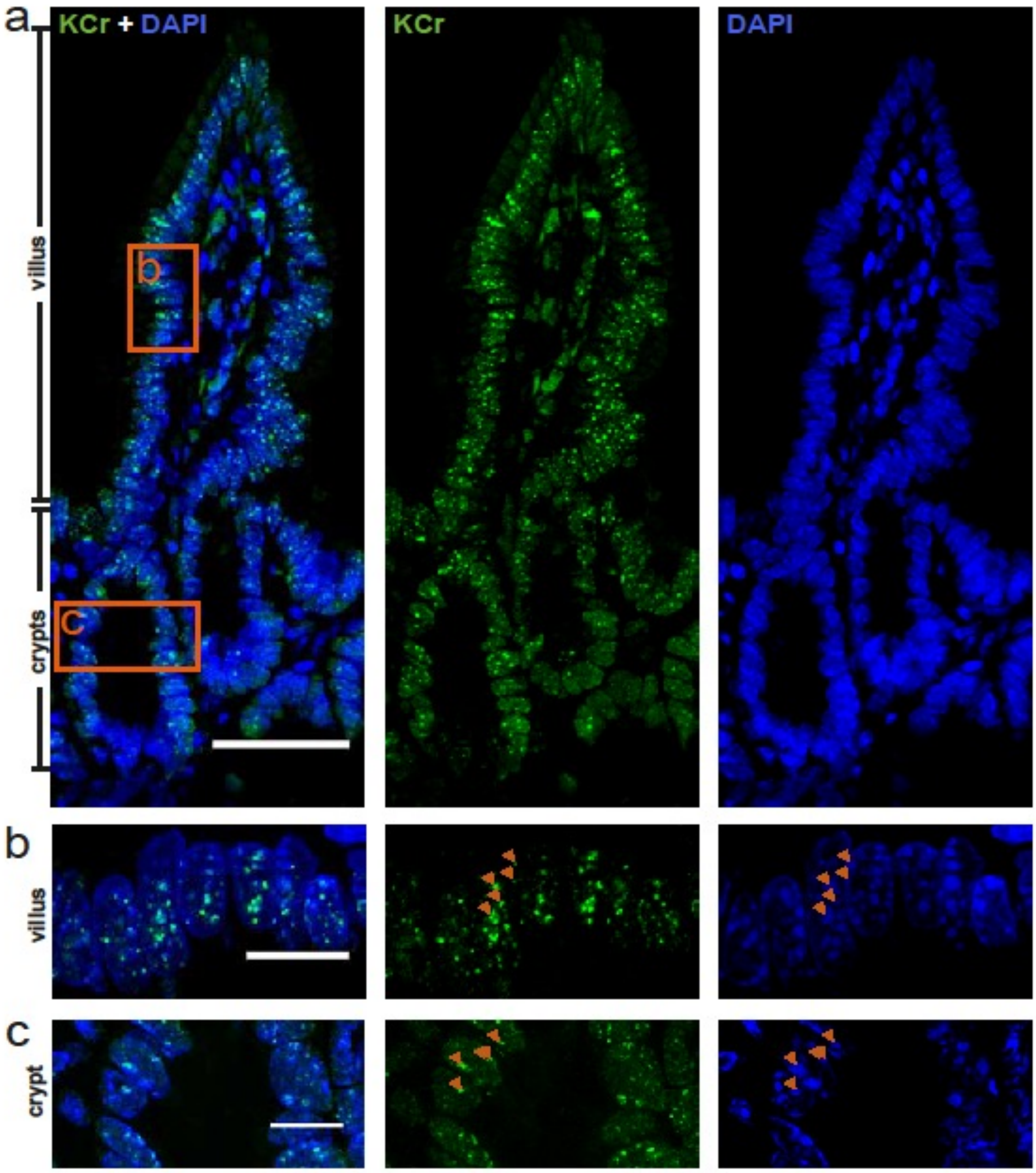

Supplementary Fig. 3. Crotonyl-lysine staining in the small intestine. (a) whole crypt/villus structure in the murine small intestine, scale bar: 40 µm. **(b, c)**: Subcellular localization of crotonylation in nuclei of villi (b) and crypts (c) from areas indicated in (a), scale bar : 10 µm. Green: AlexaFluor 488 secondary IF staining on primary pan-crotonyl antibody. Blue: DAPI nuclear staining. Arrows indicate the position of a crotonylation foci subset. Left: overlays of anti-Kcr and DAPI channels.

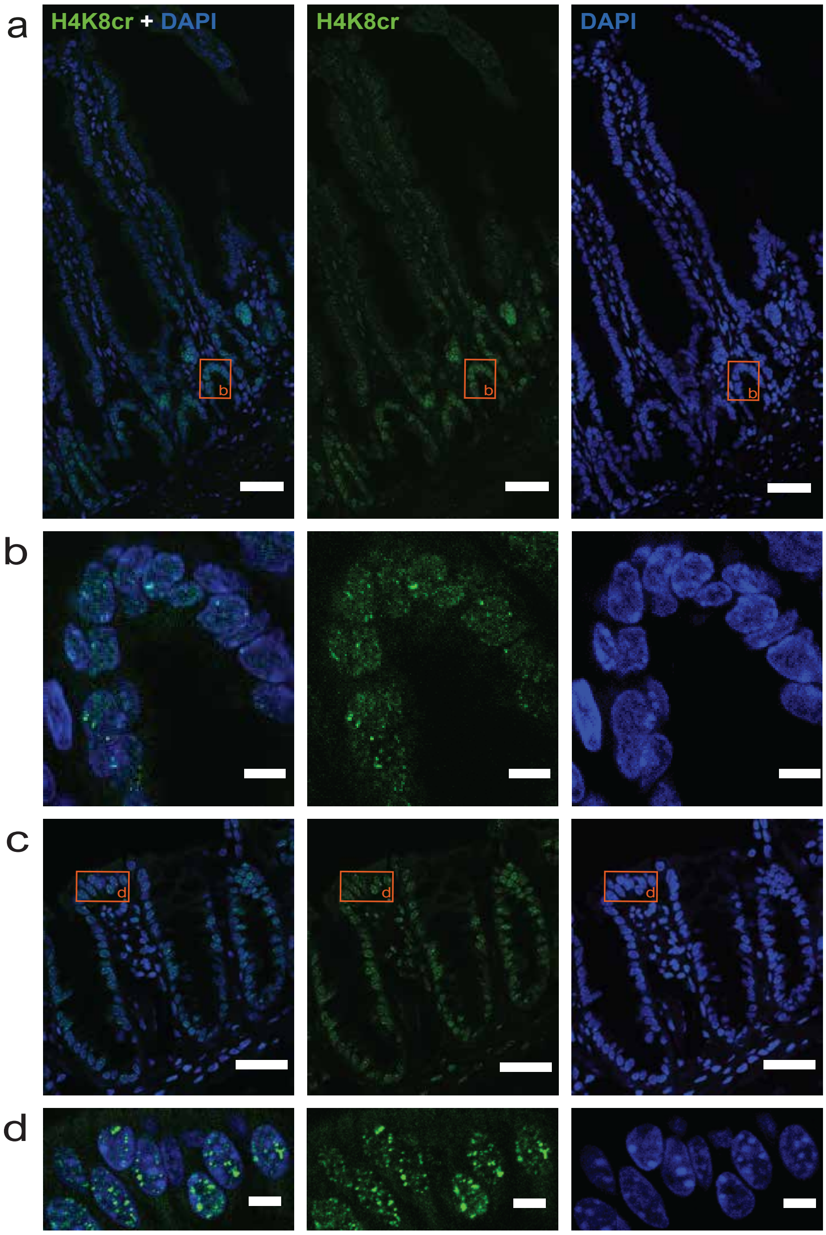

Supplementary Fig. 4. H4K8cr immunostaining in the small intestine and colon. (a) whole crypt/villus structure in the murine small intestine, scale bar: 40 µm. **(b)** subcellular localization of crotonylation in nuclei of crypts from areas indicated in (a), scale bar : 5 µm. **(c)** staining of colon, scale bar : 40 µm. and **(d)** subcellular localization of crotonylation in nuclei of colon from areas indicated in (c), scale bar: 5 µm. Green: AlexaFluor 488 secondary immunofluorescence staining on primary anti-H4K8cr antibody. Blue: DAPI nuclear staining. Left: overlays of anti-H4K8cr and DAPI channels.

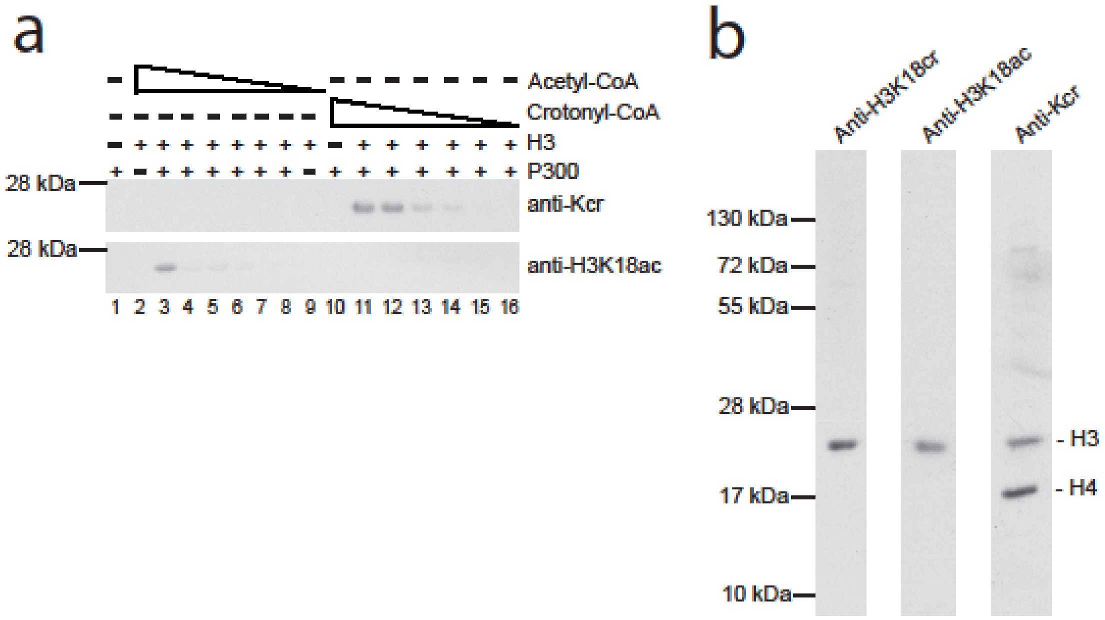

Supplementary Fig. 5. Antibody specificity test. (a) Recombinant purified histone H3 was either acetylated or crotonylated with 100 µM (lanes 3 and 11), 50 µM (lanes 4 and 12), 25 µM (lanes 5 and 13), 12.5 µM (lanes 6 and 14), 6.25 µM (lanes 7 and 15) or 3.13 µM (lanes 8 and 16) of either acetyl-CoA (lanes 3 to 8) or crotonyl-CoA (lanes 11 to 16) using recombinant catalytic domain of P300 and subjected to western blot analysis using indicated antibodies. **(b)** The anti-Kcr, anti-H3K18ac and anti-H3K18cr antibodies primarily detect histone modifications in whole colon extracts, migration positions of molecular weight markers (kDa) are indicated on the left.

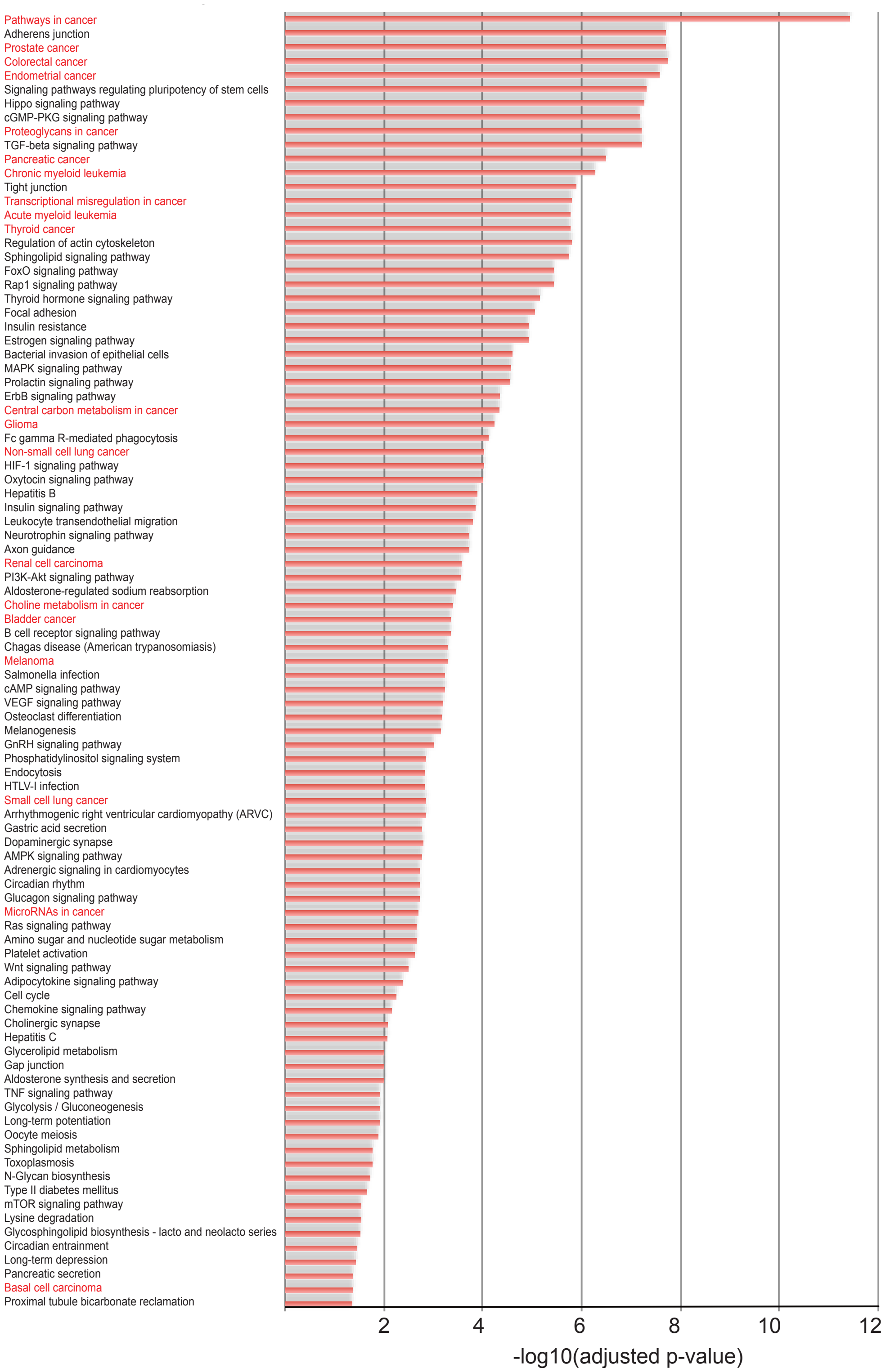

Supplementary Fig. 6. KEGG pathway analysis of H3K18cr associated genes. KEGG pathway terms for genes associated with the highest 10 percentile H3K18cr-enriched transcription start sites (TSS) in mouse colon epithelium. Cancer pathways are highlighted in red.

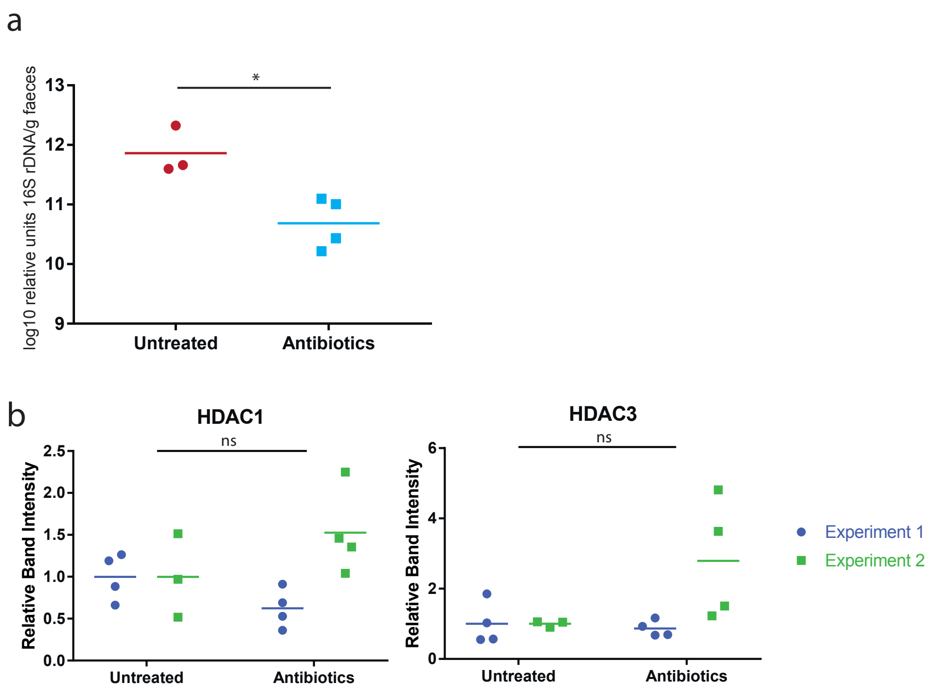

Supplementary Fig. 7. Effects of antibiotic treatment on bacterial load and HDAC1 and HDAC3. (a) Antibiotic treatment in mice led to a decrease in gut bacterial load as determined by qPCR and expressed as relative amount of bacterial DNA per gram of feaces (see Materials & Methods for experimental details). Feacal samples were from mice used in experiment 2. (**b**) Quantifications of western blot analysis of colon extracts from untreated and treated mice from two experiments. Two-way ANOVA was performed on quantified bands to compare the effect of treatment for both experiments together; ns is not significant.

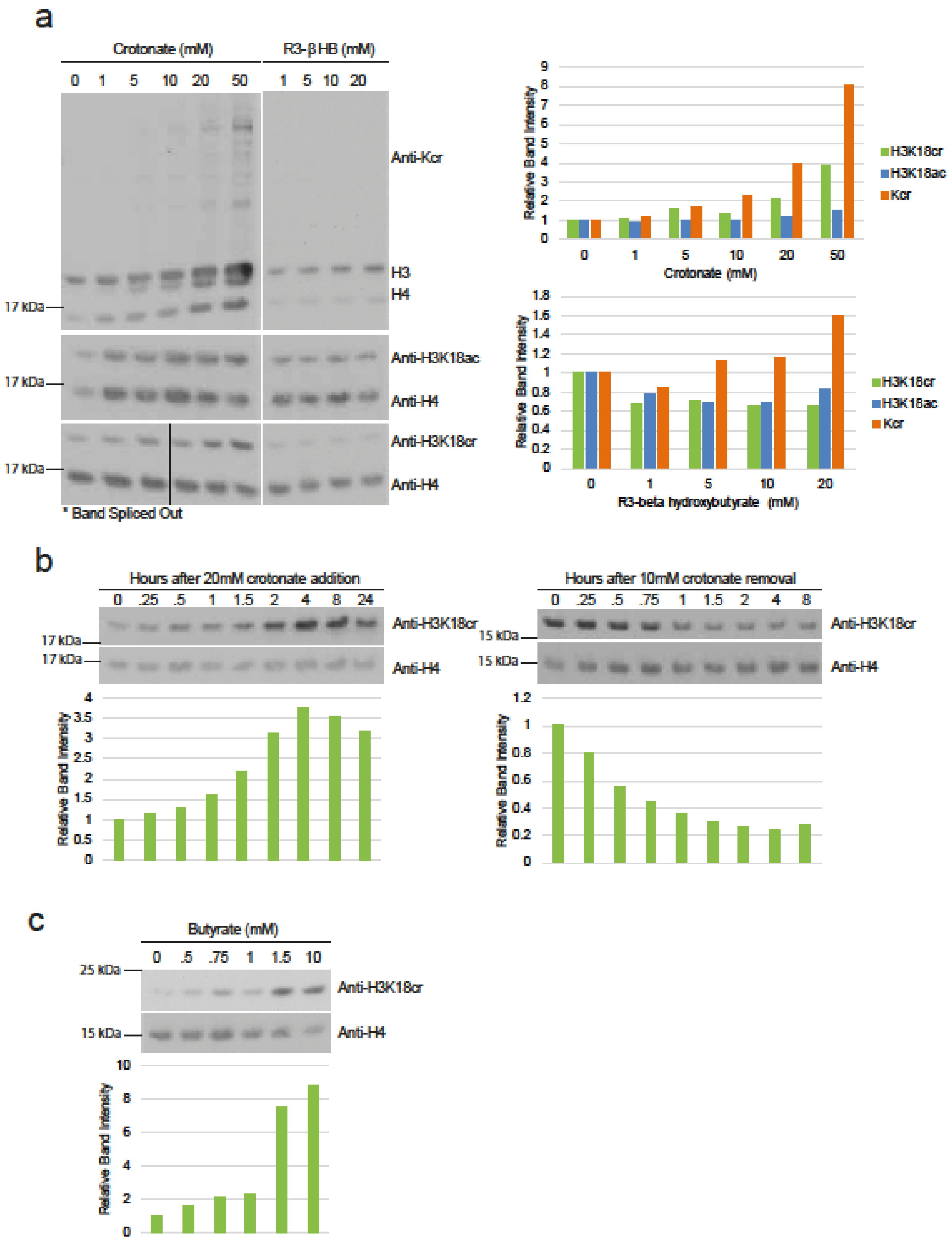
a
Crotonate (mM)
R3-β HB (mM)
0 1 5 10 20 50
1 5 10 20
Anti-Kcr
H3
H4
17 kDa
Anti-H3K18ac
Anti-H4
Anti-H3K18cr
* Band Spliced Out
Relative Band Intensity
Crotonate (mM)
H3K18cr
H3K18ac
Kcr
R3-beta hydroxybutyrate (mM)
b
Hours after 20mM crotonate addition
0 .25 .5 1 1.5 2 4 8 24
Hours after 10mM crotonate removal
0 .25 .5 .75 1 1.5 2 4 8
15 kDa
c
Butyrate (mM)
0 .5 .75 1 1.5 10
25 kDa

Supplementary Fig. 8. Crotonate and butyrate promote histone crotonylation.

(a) Addition of crotonate, but not beta-hydroxybutyrate, to the culture medium significantly promotes histone crotonylation in HCT116 cells. Extracts from cells treated with SCFAs were analyzed by western blot using the indicated antibodies with quantification on the right. **(b)** Dynamic histone crotonylation in cells treated with crotonate for up to 24 h (left panel) or for 24 h followed by wash out (right panel). Whole cell extracts were analyzed by western blot with the indicated antibodies, quantification relative to histone H4 is below. **(c)** SCFA butyrate promotes histone crotonylation in HCT116 cells. SCFAs were added to the culture medium for 24 h and whole cell extracts were analyzed with the indicated antibodies, quantification relative to histone H4 is below.

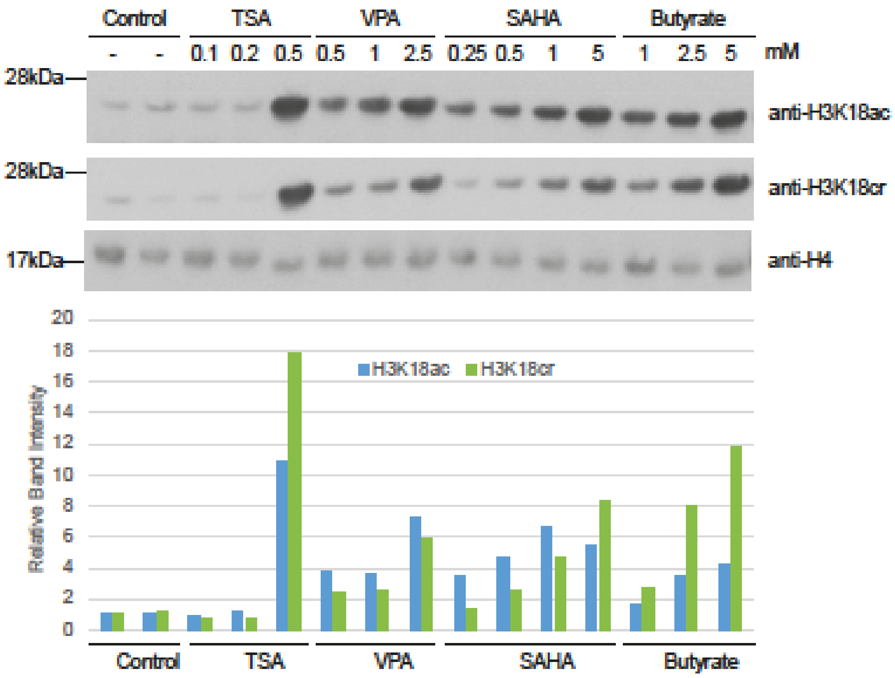

Supplementary Fig. 9. Histone crotonylation is promoted by HDAC inhibitors. Western blotting analysis of whole cell extracts from HCT116 cells cultured with indicated amounts of TSA, SAHA, VPA or butyrate for 24 h. Primary antibodies used were anti-H3K18cr, anti-H3K18ac and anti-H4. Quantification relative to H4 intensity is below.

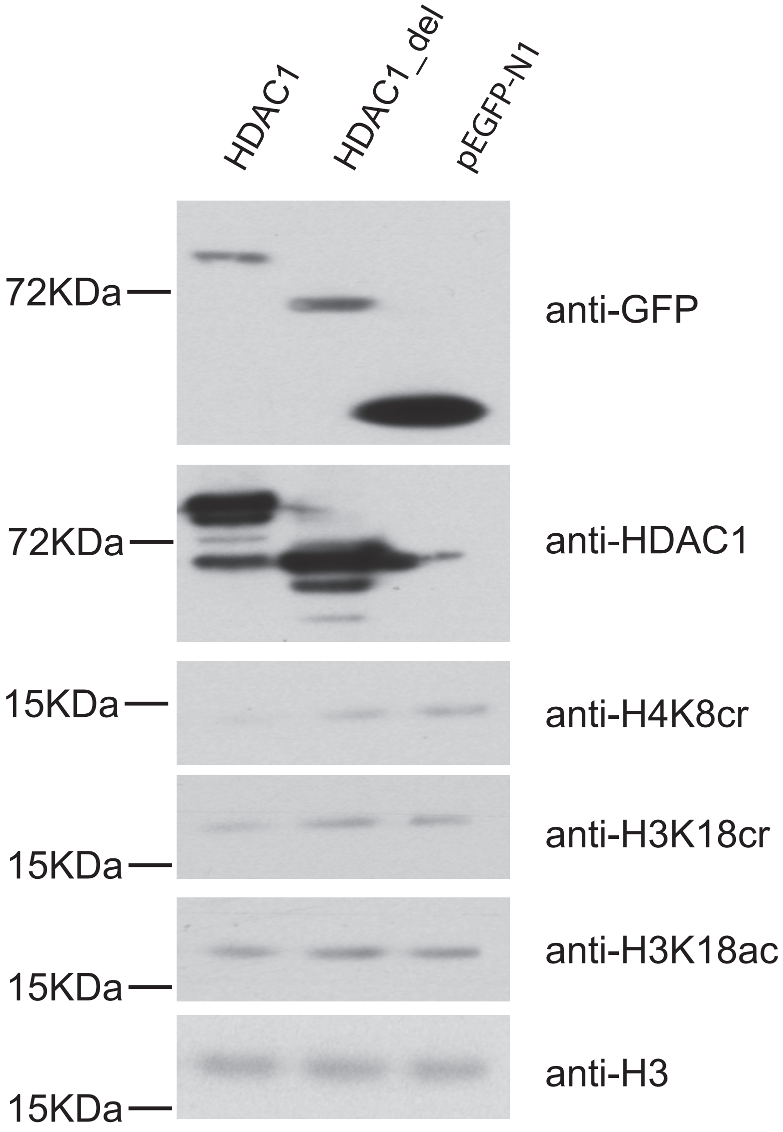

Supplementary Fig. 10. Effect of HDAC1 overexpression on histone crotonylation. Whole cell extracts from cells over-expressing wildtype HDAC1 fused to GFP (HDAC1), an 260 aa N-terminal truncation mutant thereof (HDAC1_del) or GFP alone (pEGFP-N1) were analyzed by western blot using indicated antibodies.

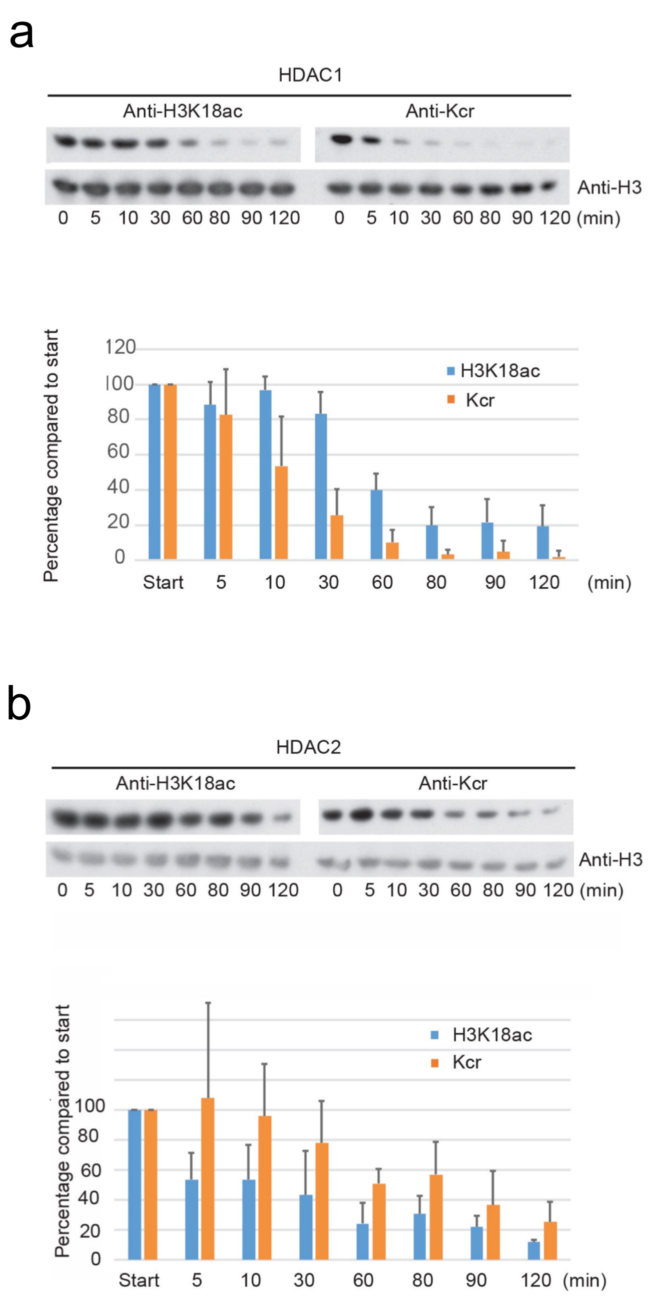

Supplementary Fig. 11. Histone decrotonylation by HDAC1 and HDAC2 *in vitro*. Recombinant histone H3 was crotonylated or acetylated *in vitro* using P300. Decrotonylation or deacetylation by HDAC1 (a) or HDAC2 (b) was monitored over time. Representative western blots are shown (no molecular weight markers were run with these gels) and the corresponding relative band quantifications (in respect to the starting time point) of three repeated experiments are below, with the band intensities for histone H3 as loading control, error bars: SEM.

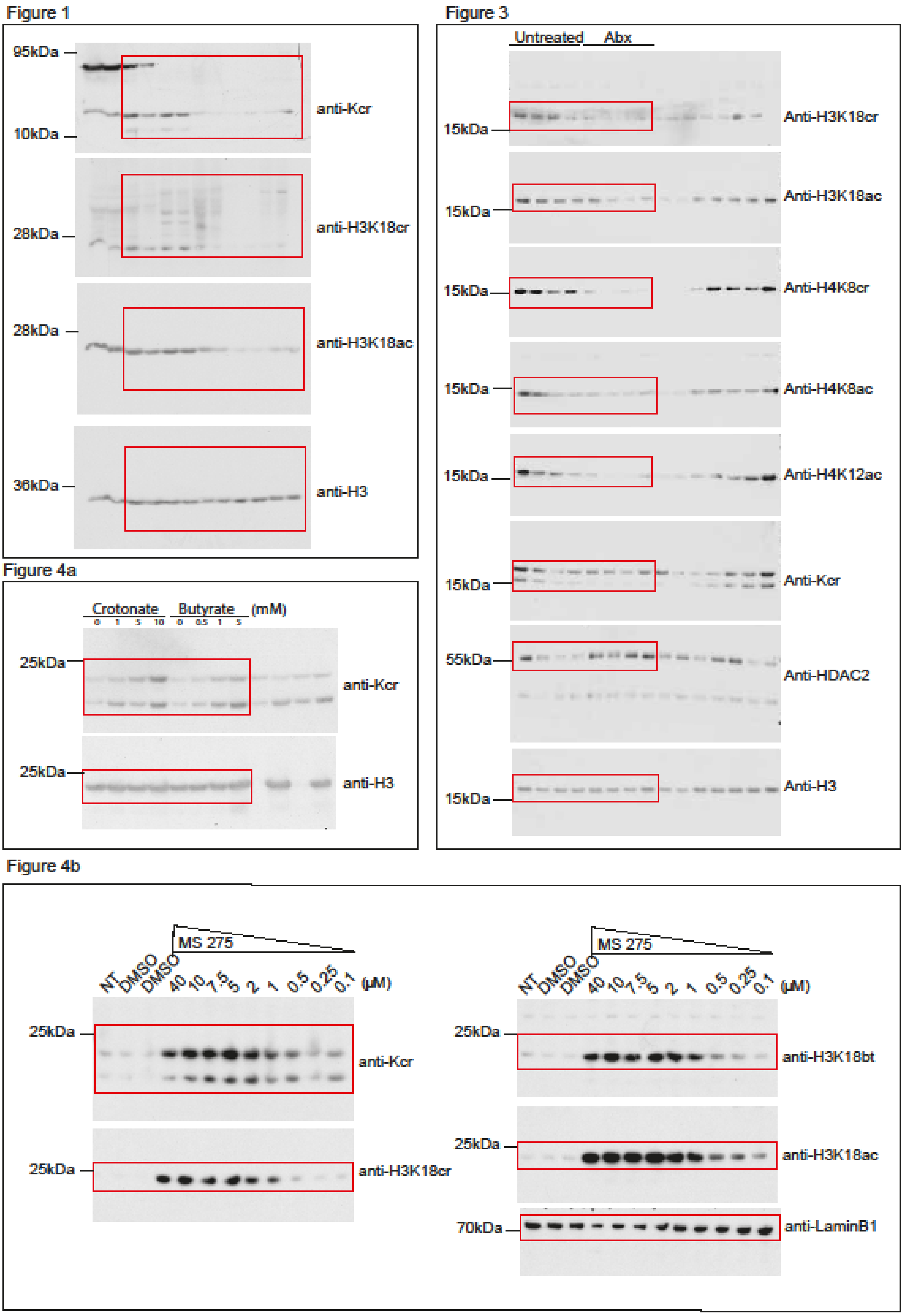

Supplementary Fig. 12. Uncropped western blots from figures 1 to 4.

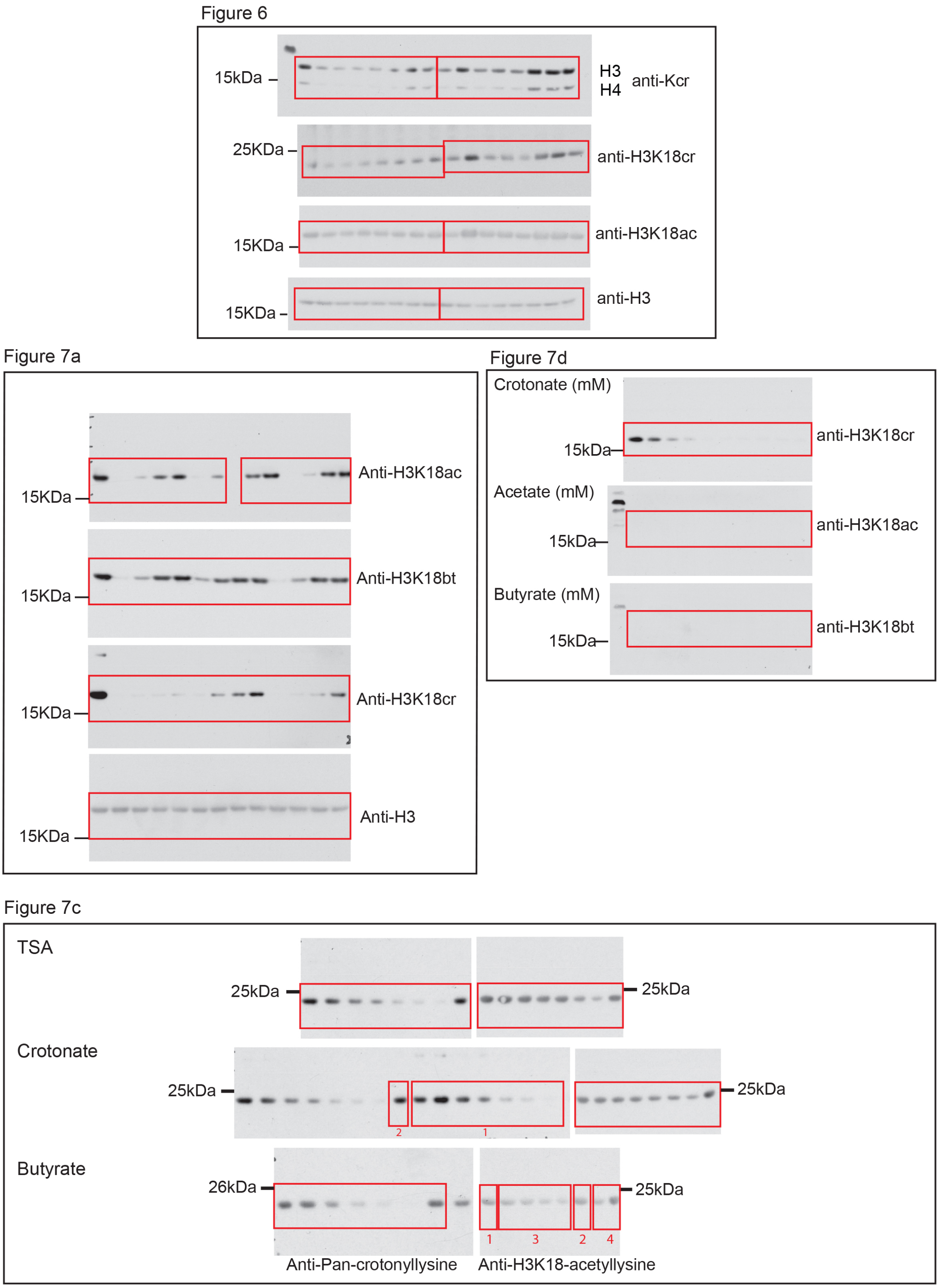

Supplementary Fig. 13. Uncropped western blots from figures 6 to 7.

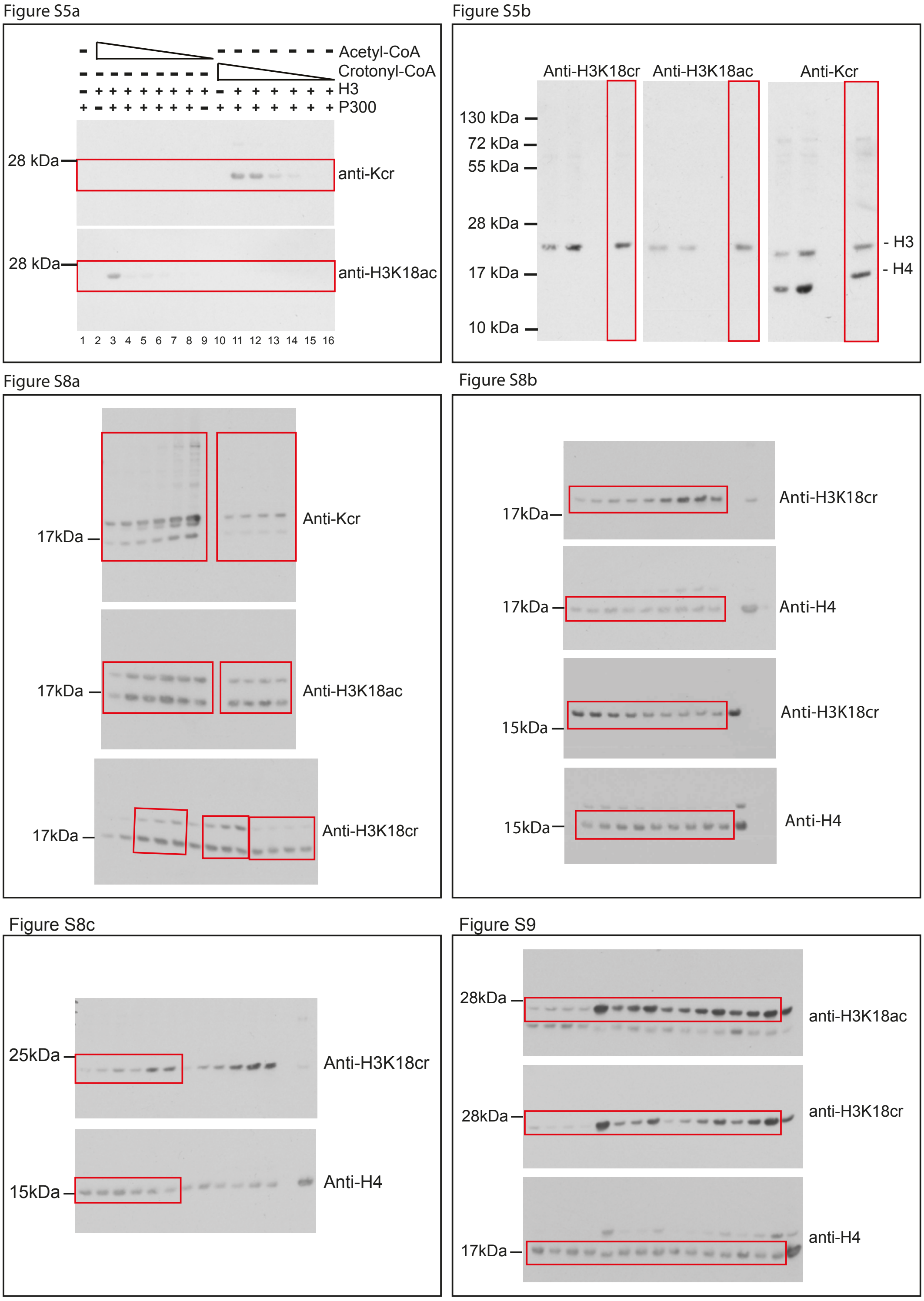

Supplementary Fig. 14. Uncropped western blots from supplementary figures 5 to 9.

a.a. Sequences	Sites	Small Intestine					Crypt					Colon					Anova Test	
		I	II	III	Median	SD	I	II	III	Median	SD	I	II	III	Median	SD	Significant	p value
H3 (3-8)TKQTAR	unmod	96.10%	96.60%	96.90%	96.60%	4.0E-03	91.80%	92.89%	92.10%	92.10%	5.6E-03	91.30%	90.80%	89.60%	90.80%	8.7E-03	+	3.8E-05
	K4me1	3.90%	3.40%	3.10%	3.40%	4.0E-03	8.10%	7.20%	7.80%	7.80%	4.6E-03	8.70%	9.20%	10.40%	9.20%	8.7E-03	+	3.8E-05
	K4me2	ND	ND	ND	--	--	ND	ND	ND	--	--	ND	ND	ND	--	--	--	--
	K4me3	ND	ND	ND	--	--	ND	ND	ND	--	--	ND	ND	ND	--	--	--	--
H3 (9-17) KSTGGKAPR	unmod	25.20%	26.00%	27.70%	26.00%	1.3E-02	21.00%	20.40%	20.80%	19.80%	3.1E-03	24.30%	24.70%	24.70%	24.70%	2.3E-03	+	1.2E-03
	K9me1	16.70%	16.00%	19.90%	16.70%	2.1E-02	12.60%	12.50%	11.60%	12.50%	5.5E-03	15.30%	15.50%	15.40%	15.40%	1.0E-03		9.3E-03
	K9me2	10.00%	9.80%	8.20%	9.80%	9.9E-03	10.40%	11.00%	11.30%	11.00%	4.6E-03	9.00%	8.50%	8.60%	8.60%	2.6E-03		7.2E-02
	K9me3	5.20%	4.20%	4.90%	4.90%	5.1E-03	4.90%	5.10%	5.40%	5.10%	2.5E-03	3.10%	3.00%	3.00%	3.00%	5.8E-04	+	6.9E-04
	K9me1/K14ac	12.00%	13.60%	11.90%	12.00%	9.5E-03	14.70%	14.30%	13.10%	14.30%	8.3E-03	16.10%	16.40%	16.30%	16.30%	1.5E-03		1.0E-02
	K9me2/K14ac	8.00%	7.60%	7.00%	7.60%	5.0E-03	10.60%	11.20%	11.50%	11.20%	4.6E-03	9.20%	8.70%	8.90%	8.90%	2.5E-03	+	2.2E-04
	K9me3/ K14ac	1.50%	1.90%	1.50%	1.50%	2.3E-03	2.90%	3.10%	3.10%	3.10%	1.2E-03	1.50%	1.50%	1.50%	1.50%	0.0E+00		1
	K9ac	20.00%	20.10%	18.30%	20.00%	1.0E-02	20.30%	20.00%	20.60%	20.30%	3.0E-03	20.50%	20.80%	20.70%	20.70%	1.5E-03		8.6E-02
	K9acK14ac	0.90%	0.70%	0.60%	0.70%	1.5E-03	2.10%	2.40%	2.60%	2.40%	2.5E-03	0.77%	0.90%	0.90%	0.90%	7.5E-04		3.9E-03
	K9cr	0.50%	0.10%	0.30%	0.30%	2.0E-03	0.50%	0.10%	0.30%	0.30%	2.0E-03	0.23%	0.10%	0.20%	0.20%	6.8E-04		6.3E-01
	K14cr	<0.1%	<0.1%	<0.1%	--	--	<0.1%	<0.1%	<0.1%	--	--	<0.1%	<0.1%	<0.1%	--	--		--
H3 (18-26) KQLATKAAR	unmod	75.31%	75.50%	75.56%	75.50%	1.3E-03	74.92%	75.30%	69.04%	74.92%	3.5E-02	63.43%	62.39%	60.46%	62.39%	1.5E-02	+	5.4E-04
	K18me1	0.37%	0.50%	0.54%	0.50%	8.9E-04	0.46%	0.47%	0.48%	0.47%	1.1E-04	0.52%	0.46%	0.56%	0.52%	5.2E-04		2.2E-01
	K18ac	23.00%	22.00%	21.80%	22.00%	6.4E-03	21.04%	21.00%	22.25%	21.04%	7.1E-03	30.86%	31.13%	31.11%	31.11%	1.5E-03	+	4.7E-07
	K18cr	0.10%	0.34%	0.84%	0.34%	3.8E-03	0.18%	0.48%	0.88%	0.48%	3.5E-03	0.22%	0.17%	0.23%	0.22%	3.1E-04		1.2E-01
	K18cr/ K23ac	0.10%	0.10%	0.10%	0.10%	0.0E+00	2.10%	2.00%	7.07%	2.10%	2.9E-02	1.34%	2.19%	2.80%	2.19%	7.3E-03		1.1E-01
	K18ac/ K23ac	1.22%	1.90%	2.00%	1.90%	4.2E-03	1.48%	1.22%	1.17%	1.22%	1.6E-03	3.84%	3.83%	5.07%	3.84%	7.1E-03	+	3.0E-04
H3 (27-40) KSAPATGGVKKPHR	unmod	24.00%	22.40%	22.90%	22.90%	8.2E-03	18.30%	17.70%	17.10%	17.70%	6.0E-03	18.60%	18.80%	18.80%	18.80%	1.2E-03	+	1.6E-04
	K36me1	1.90%	2.10%	2.00%	2.00%	1.0E-03	1.80%	1.70%	1.60%	1.70%	1.0E-03	1.70%	1.70%	1.70%	1.70%	0.0E+00		1
	K27me1	28.90%	29.70%	28.50%	28.90%	6.1E-03	24.50%	23.50%	22.20%	23.50%	1.2E-02	24.90%	24.10%	24.60%	24.60%	4.0E-03	+	9.6E-04
	K27me2	23.00%	23.00%	22.90%	23.00%	5.8E-04	30.00%	31.40%	33.20%	31.40%	1.6E-02	31.10%	31.80%	31.20%	31.20%	3.8E-03	+	5.1E-05
	K36me2	1.50%	1.50%	1.70%	1.50%	1.2E-03	1.20%	1.40%	1.50%	1.40%	1.5E-03	1.10%	1.00%	0.80%	1.00%	1.5E-03		2.7E-02
	K27me1/ K36me1	3.90%	5.00%	4.90%	4.90%	6.1E-03	3.60%	3.50%	3.40%	3.50%	1.0E-03	3.40%	3.40%	3.50%	3.40%	5.8E-04		6.8E-02
	K27me2/ K36me2	0.50%	0.50%	0.70%	0.50%	1.2E-03	2.00%	2.10%	2.00%	2.00%	5.8E-04	0.80%	0.80%	0.90%	0.80%	5.8E-04		1
	K27me3	2.00%	1.50%	1.70%	1.70%	2.5E-03	1.80%	1.90%	2.10%	1.90%	1.5E-03	1.80%	1.80%	1.80%	1.80%	0.0E+00		1
	K27me2/K36me1	8.00%	8.00%	8.50%	8.00%	2.9E-03	10.10%	10.30%	10.10%	10.10%	1.2E-03	10.70%	10.60%	10.70%	10.70%	5.8E-04	+	1.9E-04
	K36me3	0.70%	0.70%	0.70%	0.70%	0.0E+00	0.50%	0.50%	0.60%	0.50%	5.8E-04	0.40%	0.40%	0.40%	0.40%	0.0E+00		1
	K27me1/K36me2	4.00%	4.00%	3.80%	4.00%	1.2E-03	4.10%	4.10%	4.20%	4.10%	5.8E-04	3.60%	3.70%	3.70%	3.70%	5.8E-04		1
	K27me3/K36me1	0.70%	0.60%	0.80%	0.70%	1.0E-03	0.80%	0.90%	0.90%	0.90%	5.8E-04	1.00%	1.00%	1.00%	1.00%	0.0E+00		1
	K27me1/K36me3	0.90%	1.00%	0.90%	0.90%	5.8E-04	1.00%	1.00%	1.00%	1.00%	0.0E+00	0.80%	0.80%	0.80%	0.80%	0.0E+00		1
	K27cr	<0.1%	<0.1%	<0.1%	--	--	<0.1%	<0.1%	<0.1%	--	--	<0.1%	<0.1%	<0.1%	--	--		--
H3 (73-83) EIAQDFKTDLR	unmod	89.30%	89.50%	89.00%	89.30%	2.5E-03	88.70%	87.20%	87.60%	87.60%	7.8E-03	87.60%	87.20%	87.60%	87.60%	2.3E-03		7.2E-02
	K79me1	9.90%	9.70%	10.10%	9.90%	2.0E-03	10.20%	11.60%	11.10%	11.10%	7.1E-03	11.40%	11.50%	11.10%	11.40%	2.1E-03		1.1E-01
	K79me2	0.80%	0.80%	0.90%	0.80%	5.8E-04	1.10%	1.20%	1.30%	1.20%	1.0E-03	1.00%	1.20%	1.30%	1.20%	1.5E-03		1
	K79me3	ND	ND	ND	--	--	ND	ND	ND	--	--	ND	ND	ND	--	--	--	--
H3 (54-63) YQKSTELLIR	unmod	99.99%	99.99%	99.99%	--	--	99.99%	99.99%	99.99%	--	--	99.99%	99.99%	99.99%	--	--	--	--
	K56cr	<0.1%	<0.1%	<0.1%	--	--	<0.1%	<0.1%	<0.1%	--	--	<0.1%	<0.1%	<0.1%	--	--	--	--

Supplementary Table 1.

Summary of relative abundance percentage (RA%) of histone H3 PTMs detected by MS/MS at indicated peptides from histones isolated from the small intestinal epithelium, the crypt fraction of the small intestinal epithelium and the colon epithelium. We used the signal in MS1 (m/z) and the associated retention time (RT) to track their intensity and to calculate their corresponding RA% (see also Material and Methods section). The ANOVA test was applied to test significance in differences between histones PTMs from the different intestinal fractions. 'ND' indicates not-detected PTMs, likely due to low abundance. PTMs whose %RA is indicated as <0.1% were confidently identified based on the MS/MS fragmentation, but peak intensities in MS1 were too low to enable a robust %RA determination.
